# Using the behaviour change wheel to explore infant feeding peer support provision; insights from a North West UK evaluation

**DOI:** 10.1186/s13006-019-0236-7

**Published:** 2019-09-18

**Authors:** Gill Thomson, Nicola Crossland

**Affiliations:** 1 Maternal and Infant Nutrition and Nurture Unit (MAINN), School of Health, University of Central Lancashire, Preston, PR1 2HE UK; 2 School of Education, Health and Social Studies, Dalarna University, Högskolegatan 2, Falun, Sweden

**Keywords:** Breastfeeding, Breastfeeding peer support, Behaviour change, Mixed-methods, Evaluation, Infant feeding

## Abstract

**Background:**

Breastfeeding peer support is advocated in national and international guidelines, but the evidence base is mixed. In the UK, breastfeeding peer support was found to be ineffective in randomised controlled trials, while women report positive impacts on breastfeeding experiences in qualitative studies. A key criticism levied against breastfeeding peer support is the lack of theory underpinning intervention design. Here we use the Behaviour Change Wheel to structure the analysis of evaluation data from an infant feeding peer support service in one area in North West England. We aimed to provide theoretically informed insights into how peer support can be operationalised to influence women’s breastfeeding experiences.

**Methods:**

A 2 year mixed-methods evaluation (2014–2016) comprised surveys and interviews (individual or group) with peer supporters, health and community professionals, project leads and women, and routinely collected infant feeding data. We used the three layers (policies, intervention functions and behaviour-related components) of the Behaviour Change Wheel to structure and interpret the data.

**Results:**

Overall data comprised 23 interviews (*n* = 14 - individual; *n* = 9 - group) and 409 completed surveys. The findings are presented in three sections. First, the ‘policies’ (outer) layer of the Behaviour Change Wheel provides insights into the existing context, infrastructure and resources that underpinned peer support delivery. Then the second (intervention functions) and inner (behaviour components) layers of the Behaviour Change Wheel are used to present three themes, *‘developing capabilities for infant feeding’*, *‘motivating guidance and support’* and *‘opportunities for support’.* These findings highlight that a peer support service delivered in a context of effective interdisciplinary partnerships, Baby Friendly Initiative accreditation, and flexible service planning, with peer support provided via different types of instrumental, social, practical and emotional support was perceived to be highly beneficial on women’s breastfeeding experiences. In the final section key challenges faced by the service are outlined.

**Conclusion:**

While gaps and areas for development were highlighted, the service enhanced women’s capabilities, motivations and opportunities for breastfeeding. These theoretically informed insights into an organic and responsive peer support service help build the evidence base for breastfeeding peer support and to identify positive delivery features for future testing.

## Background

Breast/breast-milk feeding has compelling evidence to support its role in preventing infant and maternal ill health [1, 2]. However, despite these benefits many women do not initiate or sustain breastfeeding, particularly in high income countries such as the UK [3]. The percentages of women initiating and sustaining breastfeeding in the UK are also substantially lower within socially deprived communities; where factorssuch as marital status, fathers’ views, age, education and associated health behaviours such as obesity and smoking also linked to infant feeding choices [4–7]. The need to address socioeconomic inequities, such as through the promotion and support of breastfeeding has been highlighted in UK Department of Health (DH) guidelines and recommendations [8, 9].

Within many socially deprived communities in the UK, breastfeeding is perceived and experienced as a marginal activity [10–13]. Creating a supportive breastfeeding culture is complex and slow; however, the use of non-professional approaches, such as breastfeeding peer support schemes, is a key way in which cultural change may be achieved [14–16]. Breastfeeding peer support is provided by local women (i.e. peers) who have had their own experience of breastfeeding. The peer supporters receive training to provide informational, practical, social and emotional support [17] in groups, face to face, via the telephone, Short Message Service or via social media (e.g. Facebook) [16, 18]. Breastfeeding peer support is advocated as a supplementary means to increase breastfeeding support [19].

Overall the evidence into the effectiveness of breastfeeding peer support is mixed. Recent Cochrane reviews [20, 21] of interventions to increase the duration of breastfeeding found that skilled support, peer or professional, proactively offered to women who want to breastfeed can increase breastfeeding duration rates. However, a systematic review and meta-regression found that breastfeeding peer support interventions were less likely to be effective in high income countries such as the UK [22]. Further analysis of the trials included in the Jolly paper found wide heterogeneity in study design: a number of studies had not been implemented as planned (and were poorly reported) [23, 24]. There were also variations in regard to study participants, timing of interventions and characteristics of peer supporters [23, 24]. In contrast to the lack of effectiveness found in trial studies, a number of qualitative studies highlight how peer support is valued because it can offer women shared experiences, shared language, emotional warmth, and advocacy [10, 13, 25–27]. Peer supporters can encourage and enable access to group support (e.g. by accompanying women to groups) [13, 25], and supportive peer networks can increase social capital [16]. Qualitative studies also identify the positive impact of breastfeeding peer support on women’s confidence and capacity to continue breastfeeding [13, 14, 16, 28].

**Fig. 1:**
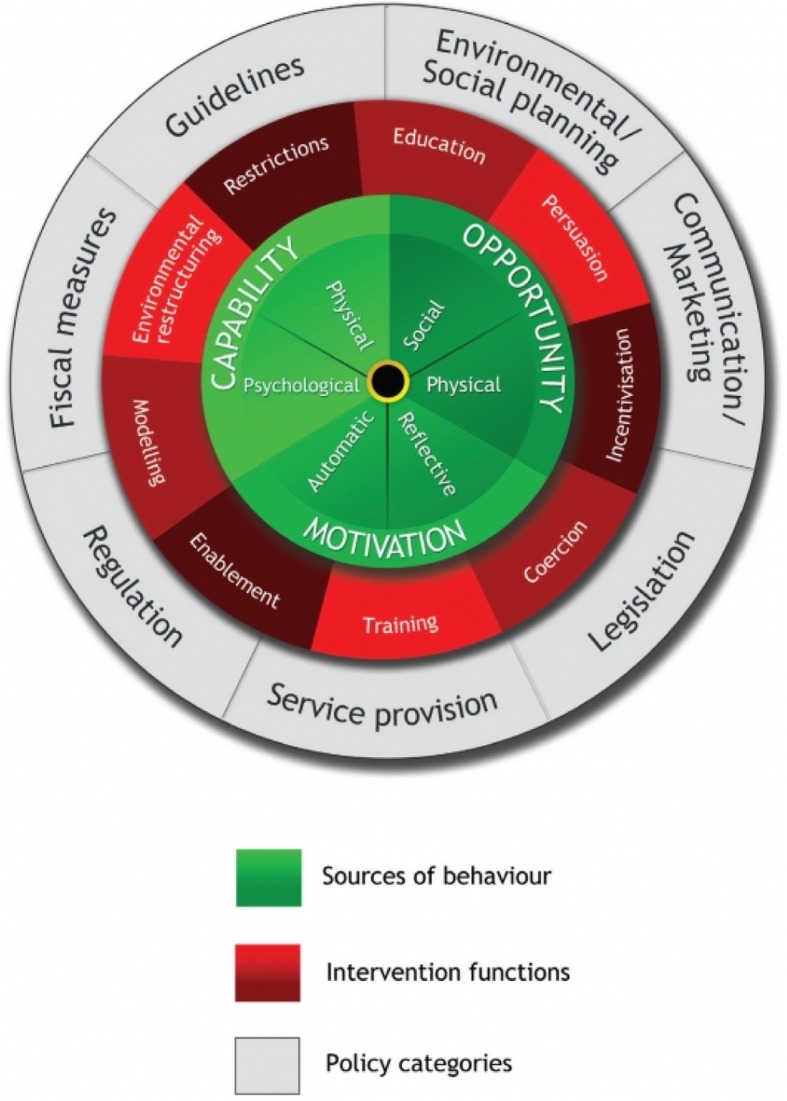
The Behaviour Change Wheel. Reproduced with permission from Michie S, Atkins L, West R. (2014) The Behaviour Change Wheel: A Guide to Designing Interventions. London: Silverback Publishing. www.behaviourchangewheel.com

While the use of theory as a framework for the development and evaluation of complex interventions is advocated by the Medical Research Council, breastfeeding peer support is a ‘notoriously under-theorised intervention’ [29]. Behavioural theories have been used to consider and identify factors that influence individual behaviours. For instance, the Theory of Planned Behavior which comprises three interlinking constructs of attitudes, subjective norms and perceived behavioural control has been used to predict [30] and explore women’s infant feeding behaviours [31]. More recently, theoretical frameworks have considered the impact of wider contextual factors on health behaviours. This aligns with a growing consensus that social, structural and resource-related issues can impact on individual behaviours [1]. One theory which considers change from a more ecological perspective is the Behaviour Change Wheel (BCW) [32], see Fig. 1. The BCW is a supra-theory model that synthesised 19 pre-existing behaviour change frameworks into a single model and comprises three interlinking layers [32]. The first (inner) layer details three essential and interlinking components for individual behaviour change: ‘capability’ (psychological, physical), ‘motivation’ (autonomic, reflective) and ‘opportunities’ (social, physical). The logic is that an individual must be psychologically and physically capable (capabilities), have social and physical opportunities (opportunities) and to be motivated to adopt specific behaviours (motivation) to enable behaviour change to occur; collectively referred to as the COM-B approach. The second layer includes nine intervention components that are designed to influence one or more of the essential behavioural components to enable positive change. These intervention functions include training, education, persuasion, enablement, restriction, incentivization and coercion. The final (outer) layer relates to policy categories that need to be in place to enable the intervention functions to operate. These include guidelines, service provision, communication/marketing and fiscal measures [32]. More simply, this model enables intervention designers to consider a) ‘what behaviours do I want to target?’ (inner layer), b) ‘how am I going to change the behaviour and what elements does the intervention need?’ (second layer), and c) ‘what needs to be in place for the intervention to be delivered?’ (outer layer). The COM-B model has been used to inform and evaluate complex interventions such as a tobacco control strategy [33] and obesity strategy [34] and to develop a motivational interviewing based breastfeeding peer support intervention [35].

Over 2014–2016 our research unit was commissioned to undertake a longitudinal evaluation of the design and implementation of an infant feeding peer support service in one location in North West England. The service comprised paid and volunteer peer supporters providing support during the antenatal, intrapartum and/or community period, in an area of low breastfeeding rates. While the BCW was not used to inform the design of the peer support service, it offered a useful analytical framework to help understand the wider infrastructure of service delivery and how the support influenced women’s breastfeeding behaviours and experiences. In this paper we do not provide a full process evaluation, but rather use the BCW wheel to structure the analysis to describe the different ‘policies’ (outer layer) that underpinned peer support delivery and how the different types and functions of support (second layer) provided by the service influenced women’s capabilities, motivation and opportunities (inner layer) to breastfeed. It was considered that this framework could add valuable insights into how peer support is operationalised to identify positive delivery features.

## Methods

### Study context

The study site is in the top 10% most deprived councils in England, with over 60% of children/young people residing in the most deprived 20% of areas nationally [36]. Approximately 25% of the population comprise minority ethnic groups. In 2014, the Public Health Department commissioned the Early Years Service (services focused on the learning, development and care of preschool children) to develop an infant feeding peer support service. Two Children’s Centre staff members who held strategic/managerial positions were appointed to lead the service, while working in close collaboration with two members of the specialist infant feeding team from the local hospital Trust. These specialist staff provided day-to-day project management of the service and trained and supervised the peer supporters. The original funds were to:

- Recruit three paid hospital infant feeding peer supporters (1.87 whole time equivalent) to provide in-hospital support, and to join an existing team of three peer supporters;
- Appoint two paid (one whole time equivalent) community peer supporters (from existing Children Centre staff) to provide infant feeding support in community/Children Centre settings;
- Recruit and train volunteer peer supporters to provide support at breastfeeding groups;
- Undertake a prospective longitudinal evaluation of the peer support service.

Formal recruitment methods were used to advertise/appoint hospital peer supporters. The community peer supporters were appointed from existing Children Centre staff whereby information on the role was issued to staff members, followed by a discussion with the Children Centre leads. Volunteer peer supporters were recruited at key points throughout the evaluation period via posters issued in hospital/Children Centre locations. All the peer supporters (paid and volunteers) were local women who had breastfed their own children. Service provision comprised an integrated antenatal, hospital and community peer support service. Antenatal support involved the hospital and community peer supporters talking to women about breastfeeding and available support at antenatal classes/clinics. Hospital support was provided by peer supporters providing daily bedside support on the postnatal ward. The community service involved the community peer supporters providing one telephone contact to all breastfeeding women (ideally within 48 h post discharge, but as soon as possible) residing in the geographical area to direct them into wider services; an early (within 48 h) and intensive proactive service that offered home visits, telephone/text support up to 8 weeks postnatal to women residing in a specific target area; and the peer supporters (community and volunteers) facilitating a number of breastfeeding groups across the locality. The volunteer peer supporters were also encouraged to sign-up local businesses (e.g. cafes, restaurants) to the breastfeeding welcome scheme in operation. All the peer supporters (paid and volunteers) received the equivalent of 2 days training and were able to access further learning opportunities via ongoing supervision/support provided by the infant feeding team. Further details on service delivery and the training/support provided to the peer supporters is detailed in section one of the findings. The main aim of the peer support service was to demonstrate an increase in breastfeeding initiation and continuation rates, with a secondary aim of promoting positive community attitudes towards breastfeeding. As the intention was for the peer supporters to also provide guidance to women who decide to use infant formula (exclusively or mixed-feeding), i.e. how to make up feeds correctly, responsive feeding, they were named ‘infant feeding’ rather than breastfeeding peer supporters. Over the evaluation period, there was a small increase in breast/breast-milk feeding initiation rates, 71% in quarter 1 2014/15 to 73% in quarter 4 2015/16, with similar increases noted within the infant-feeding discharge data (60% v. 66%) across the same period. The timeframe to collect routine breastfeeding continuation data changed from 6 to 8 weeks, to 4–6 weeks to coincide with routine postnatal check-ups with the family doctor. Therefore, while the 6–8 week ‘any’ breastfeeding continuation rates prior to introduction of the service was 34%, the 4–6 week rate at the end of the evaluation was 57% (quarter 4, 2015/16).

### Evaluation aims and design

We were commissioned to undertake a two-year evaluation of the peer support service (2014–2016) to monitor and assess take-up, attitudes and experiences. A mixed-methods study was undertaken that included surveys, interviews and routinely collected infant feeding data.

### Participant recruitment/data collection

The recruitment and data collection methods for different participant groups consulted during the evaluation are detailed as follows.

#### Peer support service members

The two Children Centre leads, infant feeding team members and hospital/community/volunteer peer supporters were invited to take part in either a group or individual interview (telephone or face to face).

#### Health/community professionals

Surveys were issued to health and community professionals (i.e. health visiting, midwifery, Children Centre staff) at two timepoints during the evaluation (~ 6 months and 18 months after project inception). At the second issue of the surveys, professionals were invited to participate in a telephone interview (and to provide contact details). Paper and online versions of the survey were issued (via email or face to face) by the infant feeding team and peer supporters.

#### Women

Opportunity sampling was used whereby peer supporters distributed different survey versions to women to capture their views and experiences of support provided at different times (i.e. antenatal, hospital, community) and by different methods (i.e. group support) – also see ‘data collection’ below. The peer supporters were instructed to give women a survey after they had been in direct receipt of support (e.g. after a discussion at an antenatal contact, after receipt of bedside/hospital support, after attendance at a breastfeeding group and/or after they had received a home visit from the community peer supporter as appropriate) together with a pre-paid return envelope. Women who received the community survey were also invited to take part in a telephone interview (through a request on the survey). It was intended that all women who agreed/provided contact information would be interviewed.

### Data collection

#### Surveys

We developed five surveys based on the Peer Support Evaluation Inventory (PSEI) (Dennis, C.L., personal communication – 10 June, 2014). The PSEI includes statements that assesses four domains of peer support – ‘supportive interactions’, ‘relationship qualities’, ‘perceived benefits’ and ‘satisfaction with support received’ – with statements scored on a scale of 1 (strongly agree) to 5 (strongly disagree). One survey was developed for health/community professionals and four surveys were designed to capture women’s views of peer support at different perinatal periods (i.e. antenatal, hospital, community) and at breastfeeding groups. We modified and/or only included statements in each of the surveys (women (*n* = 4) and health/community professionals (*n* = 1) to reflect the types of support that would be provided, and who was completing the survey. Open text comments to record benefits, challenges and recommendations were also included. Basic sociodemographic data were collected in the women’s community survey, and job roles within the health/community professionals’ surveys only. We did not collect demographic data from women who completed surveys following antenatal, hospital or group support in order to maximise completion rates.

#### Interviews

We developed interview guides to explore participants (i.e. women, health professionals, peer support staff) views, attitudes and experiences of the peer support service and recommendations for service development. The questions were developed based on the authors prior experience of undertaking research/evaluation in this area, and all interview guides were discussed and agreed by the infant feeding team (see Additional file 1). The interviews ranged from 15 to 80 min and were either audio or hand recorded (based on participant preference). On the occasion when hand recording was preferred (*n* = 1), detailed notes were undertaken.

With regard to the timing of the interviews, interviews with women were organised shortly after the completed community survey/agreement had been received; interviews with health professionals were organised ~ 18 months after project inception to allow a period of embedding to have occurred, whereas interviews with the infant feeding team/peer supporters were held at varied points to capture how the service had developed and evolved over the evaluation period.

### Data analysis

Descriptive analysis of quantitative (survey) data was undertaken using SPSS v22. All qualitative data were entered into MAXQDA qualitative software programme, with data collection and analysis undertaken concurrently. Initial analysis involved applying Braun and Clarke’s [37] thematic approach; an iterative process of identifying codes, grouping codes into sub-themes and finally creating themes that reflect the meanings and divergent viewpoints expressed. Initial analysis was undertaken by the first author, with all decisions shared, discussed and agreed with the second author. A detailed evaluation report (which includes all descriptive data) is available from the first author.

In this paper we reanalysed all the raw data (survey responses, qualitative data) using the Behaviour Change Wheel (BCW) [32] as an analytical lens. This involved reorganisation of the quantitative and qualitative data against the elements detailed within the three layers of the BCW wheel to identify: a) the different ‘policies’ (outer layer) that underpinned service delivery and b) the different types and functions of support (second layer) associated with the three behaviour-related components (capabilities, motivation and opportunities - inner layer). Any data that highlighted difficulties or challenges in the operationalisation of the service were identified and reported separately (section three of the findings). Data analysis thereby represented a modified framework approach [38], where the components of the BCW were used on a deductive basis to map and organise the data. Both authors were involved in all analytical decisions.

### Reflexivity

The first author (GT) has a psychology academic background and NC is a health researcher with a background in biological sciences. Both authors have experience of evaluating infant feeding peer support services and NC has previously volunteered as a breastfeeding counsellor for La Leche League. While both authors hold positive attitudes towards peer support, particularly in terms of its potential to enhance women’s infant feeding experiences, both believe a critical approach to optimise service delivery, and protect women’s wellbeing is needed. While GT had previously interviewed one of the infant feeding team for a different study, aside from this, the authors had no prior relationship with any of the other participants.

## Results

An overview of the qualitative data collection and survey completion for each participant group is reported in Tables 1 and 2. Overall data comprised 23 interviews (*n* = 14 - individual; *n* = 9 - group) and 409 completed surveys. We were not able to report on how many women were eligible to complete an antenatal, hospital, or breastfeeding group survey. This was due to data being collected on the number of contacts rather than individual women (e.g. numbers attending the breastfeeding groups) or including the total numbers of women who received support (e.g. on the hospital wards) which included those who did/did not reside within the geographical area of the commission. The total number of women who received home visits from the community peer supporter and therefore eligible to receive a community survey was recorded. However, the response rate is only an estimate as not all women received a survey due to ethics approval being obtained approximately 3 months after the service had started, and data collection stopping before the end of the evaluation period for write-up purposes. Overall, some 265 women received a home visit, and with 42 completed surveys received, this represents a response rate of 16%. The sociodemographic characteristics of the women who completed a community survey are reported in Table 3.

**Table 1:** Qualitative research participants

	No. participants	Data collection method
Infant feeding team	*n* = 2	Group interview (*n* = 1)
Early Years - Children’s Centre Leads	*n* = 2	Group interview (*n* = 1)
Hospital peer supporters^a^	*n* = 4	Individual interview (*n* = 2) Group interview (*n* = 1)
Community peer supporters^b^	*n* = 2	Individual interview (*n* = 1) Group interview (*n* = 2)
Volunteer peer supporters^c^	*n* = 13	Group interview (*n* = 3)
Midwives^d^	*n* = 3	Individual interview (*n* = 3)
Health visitors	*n* = 8	Group interview (*n* = 1)
Women^d,e^	*n* = 8	Individual interview (*n* = 8)

^a^ Two of the hospital peer supporters took part in an individual interview, and two took part in a focus group with the community peer supporters

^b^ Both community peer supporters took part in one group interview, and a focus group with the two hospital peer supporters. One of the community peer supporters also participated in a separate interview

^c^ Three focus groups were conducted with volunteer peer supporters

^d^ All the midwives and women took part in telephone interviews – all other interviews were undertaken on a face-to-face basis

^e^ One interview was hand recorded and all remaining (*n* = 7) audio recorded

**Table 2:** Survey participants

Participants	Responses (*n*)
Women	
Hospital	204
Community	42
Antenatal clinics	40
Breastfeeding groups	16
Health professionals	
Children’s Centre staff	61
Midwifery	22
Health visiting	16
Neonatal	1
Other	2
Not reported	5

**Table 3:** Sociodemographic characteristics of women who completed the community survey (*n* = 42)

Characteristics		*n* (%)
Age (years)	24 or under	1 (2%)
	25–29	10 (24%)
	30–34	19 (45%)
	35–39	10 (24%)
	40 or over	2 (5%)
Ethnicity	White British	25 (60%)
	Other white ethnicity	3 (7%)
	British Indian	6 (14%)
	British Pakistani	6 (14%)
	Mixed	2 (5%)
Relationship status	Married	31 (74%)
	Cohabiting	7 (17%)
	Non-cohabiting relationship	1 (2%)
	Single	3 (7%)
Number of children	1	24 (57%)
	2	8 (19%)
	3	7 (17%)
	4	2 (5%)
	Missing data	1 (2%)

Here, we present the findings in three sections. The first section, ‘operationalising and context of peer support service provision’ uses the policies (outer) layer of the BCW [32] to highlight the background context, infrastructure and resources that underpinned peer support delivery. In the second section we draw on the second and inner layer of the BCW to describe how the different types and functions of support influenced women’s breastfeeding behaviours and experiences. In the final section, ‘key challenges in peer support delivery’ we offer a more reflective account to highlight some of the challenges faced by the service, and where further work is needed.

A selection of quotes is included together with an identifier that details the participant group (e.g. mother, midwife, peer supporter), participant number, and how collected (e.g. survey, interview, focus group). When reporting on survey responses from the women, we include an identifier to indicate which survey version this relates to, e.g. A = antenatal clinics, H = intrapartum support, C = community support and G = breastfeeding groups.

### Section one: Operationalising and context of peer support provision

In this section, we draw on Michie et al.’s [32] policy components (outer layer of the BCW) to describe the wider infrastructure and context of peer support delivery. An overview is provided in Table 4, and a more detailed description provided below.

**Table 4:** Overview of key policy components

Policy category	Examples of how policies were enacted by the peer support service
Communication/marketing	• Posters in community/health venues • Leaflets distributed at antenatal/postnatal contacts • Displays on Children Centre plasma screens • Social media • Sticker on the mother’s ‘personal child record’
Guidelines	• Code of Marketing Substitutes • Infant feeding policies
Regulation	• BFI external assessment processes: accreditation in 1998, 2011, 2016 • Training/supervision provided to all peer supporters (paid/volunteers) • Peer supporter job descriptions • Regular project meetings • SMART action plans/feedback cycles
Environmental/social planning	• Home visits offered by community peer support • Breastfeeding groups at Children’s Centres • Breastfeeding welcome scheme
Service provision	• Support provided at antenatal contacts and on postnatal ward (daily support) • Breastfeeding groups • Community support up to 6–8 weeks

While the peer support service was a new area of service delivery - an integrated antenatal, hospital and community peer support service - the maternity ward, health visiting, and Children Centres in the locality had held full Baby Friendly Initiative (BFI) accreditation since 1998, 2011 and 2016 respectively. Our study focused on evaluating the peer support service rather than all infant feeding support activities taking place in the locality. However, an understanding and appreciation of the wider delivery context is essential to appreciate how the service was operationalised, and for transferability purposes.

Prior to the start of the service, the Children’s Centres, health partners and the infant feeding team had established working relationships, with a nominated BFI lead based in each Children’s Centre. Established infant feeding policies and **guidelines** were in situ, such as compliance with the WHO Code of Marketing of Breast-milk Substitutes (1981), a guideline for how breast-milk substitutes should be marketed appropriately to avoid undermining breastfeeding. **Regulation,** that is the formation of accepted practices related to infant feeding knowledge and practical support was achieved via internal audits and the UNICEF BFI external assessment processes.

All health and community staff received BFI training. The infant feeding team had developed three levels of training based on UNICEF/BFI training guidance and the nature of staff–service user interactions (Table 5). Yearly refresher training was provided, and ‘Bitesize’ information of research and evidence summaries were issued to all health and community professionals every 2–4 weeks. The infant feeding team also provided the peer supporters with additional training via focused workshops (and bimonthly newsletters) on key learning points (e.g. colostrum, skin-to-skin contact) and offered quarterly supervision sessions. All the peer supporters had the mobile numbers of the infant feeding team for immediate contact as required.

**Table 5:** Levels of training provided to health and community staff

Level	Length	Target audience	Topics covered
1	Half a day	Staff not expected to have in-depth conversations with women/families (e.g. reception, kitchen, buildings, admin).	Providing a welcoming breastfeeding environment; the benefits of breastfeeding (health and emotional); the WHO code; signposting to services/groups.
2	1 day	Staff who have in-depth conversation with women and families (but not directly in relation to infant feeding support) (e.g. Children’s Centre staff, health care assistants).	Level 1 training + how to have a mother-centred conversation; building/the importance of close and loving mother-infant relationships); responsive feeding; safe preparation of bottle feeds; co-sleeping practices; returning to work; and introduction of solid foods.
3	2 days	Health and community staff who provide infant feeding support (i.e. peer supporters, midwives, health visitors). For the volunteer peer supporters - the 2-day course was delivered over 6-weeks, 2-h/week.	Level 1, 2 + positioning and attachment; hand expression; when to refer for more specialist support; signs of successful breastfeeding; how to store breast-milk; feeding cues; support to other family members.

**Regulation** included all peer supporters having a clear job description that outlined the boundaries of support (e.g. complex or challenging cases referred to the infant feeding team, evidence-based recommendations shared only). Regular project meetings (every 2–3 months) were held between the infant feeding team, Children’s Centre leads and peer supporters. In line with standard Early Years practice ongoing feedback cycles and SMART actions plans were used to evaluate and develop service provision, which led to numerous changes over the evaluation period (discussed below).

The **communication/marketing** of the service was integrated into existing BFI-related pathways and networks such as midwifery/health visiting/Early Years meetings. Promotional activities included: posters in community/health venues, leaflets distributed at antenatal/postnatal contacts, information on Children Centre plasma screens and via social media (i.e. Facebook and twitter), and a sticker on mother’s ‘personal child record’ (book provided to all postnatal mothers to record child health and progress, e.g. weight, vaccinations). A breastfeeding welcome scheme was in operation for local public facilities; with details of venues shared via leaflets and a website. Supplementary resources such as websites (e.g. NHS Start for Life), Apps (e.g. Best Beginnings Baby Buddy App) and helplines (e.g. National Breastfeeding Helpline) were promoted and shared via multiple communication methods.

**Environmental/social planning** occurred in relation to location of support, with home visits offered by the community peer supporter, and reorganisation and expansion of breastfeeding group provision. As part of the commission, the Children’s Centres took responsibility for the delivery of three existing breastfeeding groups (previously delivered by the infant feeding team) and a further five were developed. Groups were available every weekday, at different times and in all geographical areas to coincide with other related activities (e.g. antenatal clinic, mother and baby groups).

**Service development** evolved over the evaluation period. At inception, a baseline community consultation was conducted with 25 (pregnant/postnatal) women, one father, and ~ 30 Children Centre staff to explore whether and how infant feeding support should be provided. Recommendations included the need for multiple methods of support (face-to-face, helplines, written materials), flexibly, proactively and consistently provided by trained professionals, a mass communication approach to target all women across the perinatal period, and support for fathers/partners. These underpinning messages were used to design and develop the peer support service to comprise:

- Antenatal contacts (e.g. to provide infant feeding information and support at antenatal clinics; infant feeding session to be delivered within formal antenatal education);
- Daily infant feeding support on the postnatal ward;
- Breastfeeding groups across the locality;
- Community support with the first contact provided within 48 h post discharge, and ongoing (up to 8 weeks postnatal) targeted proactive support via home visits, telephone, text; and signposting women into specialist provision (the infant feeding team) as required. The ‘expected’ intensity of contacts was alternate day visits/contact in week 1; twice weekly visits/contact in weeks 2 and 3; weekly visits/contact in weeks 4 to 8, however on each occasion, support was to be delivered on a needs-led, individual basis:
- To train/supervise volunteer peer supporters to attend/support breastfeeding groups; promote breastfeeding friendly premises scheme, and extend community reach (with 21 trained over evaluation period)

There were various changes in service delivery over the course of the evaluation. For instance, the breastfeeding group changed its format to trial a drop-in service to accommodate women who preferred not to access groups. However, as attendance at the groups markedly decreased, the groups were reinstated. Originally the two community peer supporters offered telephone support to women (referred/or identified via discharge data) and facilitated breastfeeding groups, and one also offered targeted support via home visits, telephone and/or text across the geographical area. Due to high and unmanageable numbers of referrals for targeted support, the service changed to one community peer supporter employed 30 h per week to provide support at antenatal clinics, breastfeeding groups, and proactive community support in one location only (selected due to low breastfeeding rates). The remaining community peer supporter continued to provide telephone support to women not residing in the targeted area, and to facilitate breastfeeding groups.

A further change related to in-hospital antenatal contacts. Originally the hospital peer supporters worked on a rota system to visit women on the antenatal ward (those admitted due to high-risk status). Due to difficulties associated with women’s availability and maternal wellbeing, this service changed to inviting individual women who attended hospital-based antenatal clinics (those under consultant-led care) to a 10–15-min infant feeding discussion. Prior to this change, the numbers of women seen antenatally by the peer supporters ranged from 12 to 34 per quarter in the preceding four quarters, whereas peer supporters providing contacts at antenatal clinics saw 28 to 165 women per quarter over the three quarters recorded.

A data sharing agreement was established whereby the contact details of all women breastfeeding at discharge were shared with the community peer supporters for follow up purposes. The hospital peer support service also evolved to ensure equity and all women irrespective of infant feeding method were consulted at discharge to assess knowledge and confidence and to refer to wider support as appropriate.

### Section two: developing capabilities, opportunities and motivation for infant feeding support

In this section we draw on the second (intervention components) and inner (behaviour components) layers of the BCW to describe how the different types and functions of support influenced women’s capabilities, motivation and opportunities to breastfeed. In Table 6, we map the different informational, practical, social and emotional types of support that the service provided against the three behaviour related components (inner layer) of the BCW. Each type of support could have multiple intervention functions, e.g. providing women with information on the benefits of breastfeeding involved ‘education’, ‘persuasion’ and ‘enablement’. Rather than reporting on all these separately, we list all the intervention functions associated with the types of support operating at each behaviour related component (see Table 6). Three themes are then presented which describe how the peer support service supported women in *‘developing capabilities for infant feeding’*, provided *‘motivating guidance and support’* and *‘opportunities for infant feeding support’.*

**Table 6:** Different types of support (and associated intervention functions) mapped against the three behaviour-related components

Types of support provided	Intervention functions	COM component
• Use of resources (i.e. breastfeeding dolls, visual images) to facilitate effective breastfeeding • Providing practical demonstrations (e.g. hand expressing) • Providing instrumental support to resolve women’s breastfeeding issues/concerns	Modelling Training Enablement	Physical capability
• Providing early and repeated opportunities to access information and support • Providing information that is tailored to different gestational stages • Developing women’s knowledge of infant feeding behaviours and practices • Providing information irrespective of women’s infant feeding decisions • Preparing women for infant feeding challenges and difficulties	Education Persuasion Enablement	Psychological capability
• Developing positive relationships with women • Support provided by peer supporters who understand the realities of breastfeeding • Dispelling anxieties and normalising concerns	Enablement Modelling	Autonomic motivation
• Providing information on risks and health benefits of different infant feeding methods • Encouraging women to have a ‘first’ breast feed post birth • Providing ongoing feedback and reassurance about women’s breastfeeding progress and achievements	Enablement Persuasion Education	Reflective motivation
• Breastfeeding groups offered in different localities/times • Providing flexible/proactive support • Providing women with sufficient ‘time’ to help establish/support with breastfeeding • Continuity of support between hospital and community peer supporters • Signing up local businesses to the Breastfeeding Welcome scheme • Encouraging and enabling support of partners/wider family members	Environmental restructuring Enablement Persuasion Education	Physical/Social Opportunities

#### Developing capabilities for breastfeeding

Physical capability concerns skill development *‘to engage in the activity concerned’* [32] (p.4) through feedback, demonstrations, and repetition [32, 39]. The hospital and community peer supporters used various resources to demonstrate and model effective breastfeeding, including breastfeeding dolls, knitted breasts, visual images and written resources. Breastfeeding observations and practical demonstrations, e.g. different ways to position and attach, hand expressing, breast pumps, syringe/cup feeding, helped to develop women’s skills, and offered them a range of options, should difficulties be experienced. One mother reported:

I was shown by two lovely ladies how breastfeeding worked i.e. stimulation, hormones, when milk comes, what to do if I’m struggling to get baby to latch. I was shown how to express into a syringe as my daughter was being fussy, this was so helpful and most of all I have felt so relaxed and comfortable letting them do what's been needed to get me from A to B (Mother, no. 185, hospital survey).

The resources and skill-based support from the peer supporters were reported to have helped women to resolve pain, achieve an effective latch, e.g. ‘*she helped me to latch my baby on correctly’* and to *‘effective express’.* While between 93 and 100% (C, G, H) of women agreed to the statement  that the peer supporters had helped them to ‘resolve my [breastfeeding] problems’, almost all women (93–98%, A, C, H) agreed that they felt ‘more able to solve problems or concerns about breastfeeding’. These insights thereby indicating how the peer support served to empower women to develop their own capabilities to breastfeed:

When she first came to see me, I was tired and frustrated and feeling guilty that my baby always seemed to be unsatisfied after her feeds. She put my mind at rest and helped me to relax. She visited me two more times after this until I had a complete turnaround and felt like I was succeeding (Mother, no. 34, hospital survey).

*Psychological capability* relates to knowledge and understanding; how knowledge is shaped by enabling interventions [32, 39]. The peer supporters offered an important early intervention service to ‘shape’ women’s knowledge about breastfeeding. They referred to how they would *‘plant seeds’* (verbally and/or written) to increase women’s understanding about the nature, benefits and practicalities of breastfeeding during the antenatal period, with this information then reinforced during postnatal contacts. The information included benefits of colostrum, skin-to-skin contact and feeding cues and patterns. One peer supporter emphasised that rather than a focus on the practical or technical approaches of breastfeeding, it was important to transmit an understanding of infant behaviours to parents:

Make them [parents] see it [breastfeeding] a different way, why babies are feeding a lot, every three hours, why that isn’t necessarily right (Hospital peer supporter, no. 1, interview).

Between 95 and 100% (A, C, H) of women agreed that the peer supporters had led them to feel ‘more knowledgeable about breastfeeding’, with qualitative comments confirming how the support had provided new information and understanding:

Breastfeeding was always explained as the food side for the baby. It was a nice insight to know about the emotional bonds that the baby enjoyed, closeness to the mother and the amount of milk they need especially the size of their stomach (Mother, no.5, hospital survey).

Women valued knowing the ‘science’ of breastfeeding, and which could lead to women having more options that they thought available:

Finding out that I can double breastfeed my twins at the same time and if I wanted to breastfeed my other child at the same time (Mother, no.11, antenatal survey).

One hospital peer supporter referred to how she would impart breastfeeding information irrespective of the parents’ receptivity. This reflected a persuasive strategy that was considered essential to ensure *all* women received breastfeeding-related information and to make a fully informed choice:

You will get the ones that don’t, you know, they just think I’m butting in [by providing information on breastfeeding] or what have you. But I am not going to sacrifice people who really do want to know with those who don’t, you know, they’re a minority (Hospital peer supporter, no. 2, interview).

Repeated antenatal contacts (at hospital/community antenatal clinics) meant tailored information being provided at different gestational stages, e.g. an early introduction to health benefits and bonding during the first trimester, and how to deal with breastfeeding difficulties and where to access support discussed during the third trimester. Antenatal contacts helped to raise awareness about potential challenges, such as ‘*unsettled nights at the beginning*’ and to prepare women mentally, physically and psychologically to breastfeed:

I knew nothing at all about breastfeeding with this being my first baby. I have learnt a lot about the benefits, best positions to feed and what is normal. This has made me very calm, confident and positive to continue to breastfeed for as long as necessary (Mother no. 43, community survey).

Overall 72% of women responding to the hospital survey and 88% of those responding to the breastfeeding groups survey agreed that they would 'not have been able to continue breast/breast-milk feeding without the help of the peer support service', with qualitative comments echoing this sentiment, e.g. *‘If I hadn’t had this amazing support, my baby would have been on formula after three days’*:

I feel this service is invaluable to women who, like me thought breastfeeding was a natural thing that just happened. There is far more to it and without the help of those amazing ladies, I would have given up and not been able to give my newborn baby the best start in life, SO thank you, eternally grateful (Mother no. 142, hospital survey).

#### Motivating guidance and support

*Autonomic motivation* concerns emotions and responses that arise from associative learning [39]. In this context, this relates to how women feel and respond when learning to breastfeed via interactions with the peer support service. We highlight how the qualities of the peer supporters and nature of the peer-woman relationship helped to enhance women’s positive responses towards breastfeeding.

Opportunities for the peer supporters to build relationships with women were considered crucial. Since 2012, the UNICEF UK Baby Friendly Initiative standards have emphasised the importance of mother-infant relationships [40], and a relational approach to breastfeeding support. All the peer supporters had been trained in and used a relationship building approach that included open, non-judgemental, active listening and positive interpersonal behaviours (eye contact, facial expressions and body language). Almost all women reported positive experiences of the peer supporters, using terms such as ‘*helpful*’, ‘*understanding*’, ‘*kind*’, ‘*lovely*’, ‘*patient*’, ‘*friendly*’, ‘*caring*’, ‘*reassuring*’, and ‘*supportive*’ to describe them. The proactive and regularity of contact with the peer supporters as well as their thoughtful gestures, e.g. ‘*took time out of her day to accompany me to NICU’* provided women with a sense of being cared for. The positive nature of the peer–mother relationships is reflected in 98% (C) agreement by women that the peer supporters ‘accepted me for who I am’ and how they knew the peer supporters ‘would respond to me in a supportive way’. One of the women who received support from a hospital-based peer supporter reported:

I found peer supporter very helpful and understanding. She showed the same understanding and concerns as a normal mother and gave advice as we went along. If I had a choice I would have taken her home with, that’s how caring and supporting/knowledgeable she was (Mother no. 140, hospital survey).

Similar to insights within the wider literature [13, 41] women often described their relationship with the peer supporters as a ‘friendship’, *‘she felt like a friend I’d known for years*’. Women valued receiving support from mothers who understood the realities of breastfeeding, who ‘*knew what I was talking about’*, and which in turn helped to facilitate a positive women-peer supporter connection, ‘*I felt she* [peer supporter] *could relate to me a lot more*’*.* Within the community survey, 95% of women agreed that the peer supporters had understood their experiences, and how they were feeling. The importance of the peer supporters first-hand experience of breastfeeding was also noted by health professionals, as illustrated below:

Enables women to build a relationship with somebody who has had personal experience. Promotes confidence in women and families and enabling them to have more informed choice and knowledge (Health visiting professional, no. 13, Health/community professional survey 1).

The peer supporters also referred to how an empathic approach and open dialogues encouraged women to offload concerns and to share experiences and learning:

Listening to how they feel really. Helping them when they need help, answering questions when they need questions answering. Being honest with them, giving them advice on what they need advice on. And I think it just comes over time, the more contact you’re in with that person, that relationship builds up and they know that they can, they know you’re there if they need you, because you tell them that (Community peer supporter, no. 1, interview).

One of the key means to help motivate women to continue breastfeeding was through dispelling anxieties and normalising their concerns. From the survey responses between 95 and 100% of women (C, G, H) agreed that the peer supporters had helped them to feel that ‘what I was going through was normal’, with similar sentiments reflected in the qualitative data:

I was very anxious and worried that I had done something wrong or there was something wrong with my baby. X [peer supporter] made me see it was just a setback and we would be fine, her continuous support was invaluable and reassuring (Mother no. 59, hospital survey).

Women frequently referred to how the peer supporters had helped alleviate a sense of pressure and blame, with 93–99% (A, C, G, H) of mothers agreeing that the support had led them to feel ‘less worried’. The peer supporters reassured women they were *‘doing it* [breastfeeding] *right’*, there was *‘no set routine’* for breastfeeding, and that breastfeeding can *‘take time’* for mothers and infants to learn together*.* One woman stated:

I’m actually starting to get a bit emotional now thinking about it. She was just so reassuring. I remember once her ringing, she rang me and I was crying because I was so upset about it all, and she was just like, you’re doing the right thing, stick with it, you know, everything you’re doing is right, you’re doing really well. And just someone telling you you’re doing the right thing is so reassuring and comforting. Because, obviously, you’re emotional anyway, and when things aren’t going right, it just makes everything worse (Mother no. 6, interview).

*Reflective motivation* relates to conscious decision-making such as through evaluations and plans [32, 39]. This aspect believed to be improved through techniques such as providing information about consequences, setting goals and improved self-belief [32, 39].

Reflective motivation was enhanced during antenatal contacts through the peer supporters introducing and discussing risks and benefits associated with different infant feeding methods. While some of the mothers had already intended to breastfeed, this information could still be empowering as it reinforced their beliefs and motivations, ‘*confirmed our thoughts that breastfeeding is the right choice for us’*. Whereas for those who held a more ambivalent attitude, this information was reported to have helped debunk myths and to have positively influenced their beliefs towards breastfeeding:

I learnt so much about the benefits and prior to this was unconvinced whether I would bottle or breastfeed – I had no doubts after the all the advice and support from the [peer support] team (Mother no. 20, antenatal survey).

From the survey responses, between 84 and 100% (A, C, G, H) of women agreed that they felt ‘more determined to breastfeed’, which for some led them to envision their breastfeeding goals:

Advised benefits to carry on breastfeeding till age of two which I wasn't aware of so will definitely try and breastfeed as long as possible (Mother no. 70, antenatal survey).

A powerful means to influence women’s reflective motivation to breastfeed was via encouragement to provide a ‘first [breast] feed’ post birth. The peer supporters reflected how antenatal encouragement had led to more women willing to *‘give it a go’* and how the positive experience had facilitated breastfeeding continuation:

There are far more women that have had those antenatal conversations, either with our team or the volunteers that work the antenatal units. They have done the skin to skin and [mother] said, well I’ll do a first feed, and quite often, you know, I meet women a lot on the ward who are now on day two or day three and it’s going really well. (Hospital & Community peer supporters, focus group)

Peer support was reported to have directly impacted on women’s self-efficacy to breastfeed, ‘*I am now really happy and comfortable with the twins feeding and able to enjoy the bonding’*. Peer supporters provided ongoing feedback (with between 97 and 98% agreeing to this statement in the community and hospital surveys) on both a physiological (e.g. ‘*she told me I had got it* [latch] *right’*) and emotional (e.g. ‘[peer supporters showed] *genuine delight and encouragement at successful feeds)* basis*.* Overall, between 88–99 % (A, C, G, H) of mothers agreed that the support from the peer supporters had led them to feel ‘more confident’ in their abilities to breastfeed, and as reflected in the following quote, to seek out further support as required:

The peer supporter normalised my problems/concerns which gave me the confidence to ask more questions, persevere with breastfeeding and greatly reduced frustrations that may have otherwise put me off continuing to breastfeed (Mother no. 86, hospital survey).

#### Opportunities for infant feeding support

Opportunities concern physical and social factors that lie outside the individual that can help to prompt and encourage behaviour [32, 39]. While these feature as separate factors in Michie’s model, it was less helpful to perceive them as individual components, as physical opportunities can inevitably encompass social encounters.

The peer support service offered a transition of flexible and proactive physical and social opportunities to access support across the perinatal period. The service included antenatal information and education, in-hospital support, early and prolonged community support for those within targeted areas; and access to more specialist (i.e. lactation consultant) and wider support (e.g. helplines, social media, groups, health visitors, midwives) as needed. Overall, between 98 and 100% of women (A, C, H) were aware that help was available should they need it, and 94–98% (C, G) of women agreed that the peer supporters had helped them to access other support services in the locale. Some 97–100% of women (C, G, H) reported that they ‘felt less alone through knowing that wider support was available’. Being able to access practical and/or emotional support as needed provided women with the reassurance of a *‘comfort blanket’ –* a *‘lifeline’:*

My child had a tongue tie and sometimes didn't latch on very well, having the support helped me to know that it was doing things correctly. Just knowing there was someone to talk to really was a good support when I was feeling uneasy (Mother no. 15, community survey).

The opportunities for support were also temporal, peer supporters had time to spend with women, to listen to their concerns and provide needs-based care, with between 98 and 100% (C, H) of women agreeing that the peer supporter had invested sufficient time to help support infant feeding. From the qualitative data, healthcare providers, peer supporters and women referred to how embodied time to listen to concerns, resolve issues, and provide support when needed was crucial for successful breastfeeding, with one of the midwives reporting ‘X [peer supporters] *had time which for a new mum was absolutely invaluable’.*

Opportunities for continuity of care from the antenatal to intrapartum period were offered where possible. Community peer supporters would notify hospital peer supporters about women who would benefit from additional support, e.g. a mother who did not want her breasts being touched, or to reinforce women’s motivation instilled through antenatal discussions:

I did send an email to X [infant feeding lead] saying she’s [woman who initially did not want to breastfeed] coming in soon, can you watch out for her. Because she was adamant she didn’t want to [breastfeed] but now she’s got into it after having this discussion (Hospital and community peer supporters, focus group).

Close communications between hospital and community peer supporters also enabled timely postnatal support to be provided:

A call came in [from the hospital peer supporters] and X [community peer supporter] could go which was brilliant, and that was all this woman needed, someone to sit, and watch her feed. (Community peer supporter, no. 2, interview).

Eight breastfeeding groups were established to ensure a group was available every weekday, at different times and in all geographical areas. The groups also coincided with other related activities (e.g. antenatal clinic, mother and baby groups) to facilitate ease of access, encourage attendance, and promote access to wider Children’s Centre activities. Some 81% of women who had accessed the breastfeeding groups agreed that these events had helped them make new friends, with qualitative comments reflecting how the groups offered a support network for breastfeeding and maternal wellbeing:

Rather than just a one-off visit it has created a longer-term relationship not just with the support worker but with other mothers as I was encouraged to attend breastfeeding support groups. I feel like there is a support network to help me (Mother no. 30, community survey).

The volunteer peer supporters were also involved in signing local businesses up to become ‘breastfeeding friendly’ with information distributed to women via the peer supporters and a local website. Some of the mothers who had received this information, referred to how this had boosted their confidence to breastfeed outside the home environment:

She gave me loads of information about places locally that I could go and feed [baby] without having to necessarily purchase anything. You can just go in, feed, and they’re quite comfortable for you to do that. That was a big help. (Mother no. 2, interview).

The influence of partners and wider family members on women’s infant feeding decisions and experiences is well reported [42–44]. The peer supporters could experience opposition and challenges such as through partners or grandparents wanting to feed the infant, or the perceived value of formula, *‘*[In] *India and Pakistan, the well-off people choose to give formula milk because it is* [high] *status*’. All peer supporters referred to how they would make concerted efforts to engage with fathers and wider family members to maximise women’s opportunities for physical and social support to sustain self-feeding*.* This involved providing information to help change attitudes and knowledge of infant feeding and offering suggestion as to how support could be provided, *‘so* [tell partners] *make them food, do the cleaning, things like that’*. The peer supporters referred to how a sensitive approach was important in these encounters, to value perspectives while *‘carefully’* offering counterfactual information:

And it’s about doing that in a really gentle way and [saying to partners/family members] actually, as time’s gone on we’ve learnt things and we’re discovering new things all the time. And the current research tells us that, you know (Hospital and community peer supporters, focus group).

Some women specifically highlighted how the peer supporters had enabled their partners to be more supportive, pro breastfeeding and confident in breastfeeding:

Made my husband much more confident in breastfeeding, the reasons why we are breastfeeding and provided a second thought for when giving up may be the easiest option. (Mother no. 190, hospital survey).

### Section three: key challenges in peer support delivery

Areas where women did not receive support to develop capabilities, opportunities and motivation for breastfeeding, and those suggested for further development of the service were highlighted during the evaluation. First, there could be difficulties in meeting women’s needs. As the majority of peer supporters were White-British, it was not always possible to provide culturally appropriate support. A few health professionals and women also reflected the need for more support with formula feeding mothers and how an overzealous breastfeeding approach instilled guilt and blame, rather than motivation:

It was a little too pushy on breastfeeding only which with a new baby can make you doubt yourself and feel bad about your decisions rather than reassure you. (Mother no. 107, hospital survey).

Some women faced difficulties in accessing group based support due to its location, e.g. *‘one mum had to get on a couple of buses to get here’,* and how some mothers *‘are not group people’* and lacked confidence in entering an unknown/public forum. This was felt to be especially challenging for mothers who had breast/breast-milk feeding difficulties and those from specific demographic groups, e.g. *‘young mums’*. These examples illustrate how some women did not experience some of the ‘opportunities’ for breastfeeding support that the service offered.

Time pressures and restricted resources also limited women’s opportunities to access support. Meaningful, needs-led breast/breast-milk feeding support is a time-inducing endeavour, and all peer supporters disclosed personal and logistic challenges in providing ‘*quality’* care. This issue was compounded in the hospital environment, by the complexity of infant feeding issues experienced, with the peer supporters having to triage as needed*.* Targeted community support provided by one individual was also considered inappropriate and unsustainable, ‘*so if she* [peer supporter] *goes down, then the service goes down*’. Furthermore, while ‘some’ continuity of carer was provided through the peer supporters providing antenatal and intrapartum/postnatal care to the same woman, this was ad-hoc and unplanned, potentially missing occasions to develop women’s capabilities, and to sustain their motivation.

## Discussion

In this paper we used the Behaviour Change Wheel (BCW) [32, 39] to structure the analysis of the findings from an evaluation of an infant feeding peer support service. The ‘policies’ (outer) layer of the BCW helped to illuminate the existing context, infrastructure and resources that underpinned peer support delivery. We also used the second (intervention functions) and inner (behaviour-related components) layers of the BCW to identify and describe how the informational, instrumental, social and emotional types of support influenced women’s capabilities, motivation and opportunities for breastfeeding. Women’s physical and psychological capabilities were developed through practical support and demonstrations and information on breastfeeding behaviours. Women’s motivation was enhanced through positive, trust based relationships formed with peers who understood the realities of breastfeeding, educating women on the health benefits of breastfeeding, and ongoing feedback on women’s achievements. Opportunities related to a framework of flexible and proactive support that spanned the perinatal period and extended its reach from hospital and community settings to wider public venues. The peer supporters also engaged with wider family members where possible to engage and enlist their support to help women sustain breastfeeding.

### Policy implications for peer support delivery

The policies layer of the BCW enabled us to specifically consider the wider context, resources and practices to underpin peer support provision, detail that is often lacking in published articles. In this service, peer support delivery had evolved from within a context of an existing hospital-based peer support service and good interdisciplinary collaboration between health and community services. Wider literature identifies how positive partnership practices and interdisciplinary service collaboration are needed to ensure that women and families can benefit from a wide range of expertise [45], and effective integration of peer support with healthcare pathways has been identified as an effective feature of breastfeeding peer support [29]. All the health/community areas had full BFI accreditation, and had adopted an inclusive approach to training, thereby mitigating against women receiving inconsistent infant feeding information which women perceive negatively [46, 47]. The service utilised wide and varied promotional methods, in line with women’s preference to receive information from a variety of sources [48, 49]. The organic, flexible and responsive nature of service development and delivery, via community consultations and evaluation cycles, also served to ensure that service provision met service-user needs, with an approach that is advocated in research and policy [28, 29, 50]. However, the evident gaps in the service meeting the needs of certain population groups indicates that further work is needed for equitable support provision.

### The influence of peer support on women’s capabilities, motivation and opportunities

Many of our findings in regard to how peer support can influence women’s capabilities, motivation and opportunities for breastfeeding parallel the elements of effective breastfeeding peer support described in the realist review by Trickey et al. [29], and in other studies [51] or systematic reviews of breastfeeding support [21]. For example, Trickey et al. [29] and the recent multicentre RUBY (Ringing Up about Breastfeeding Early) trial undertaken in Australia found that repeated contacts in the early postnatal period (a risk period for discontinuation), and proactive support, created important opportunities for breastfeeding support [52]. Furthermore, our findings emphasise how emotionally warm and enabling relationship with the peer support enhanced women’s motivation, similar to the elements of emotional accessibility identified by Trickey et al. [29] and others [35, 51]. Our findings support wider literature that emphasises the need for multiple opportunities for women to access support for infant feeding [13, 16]. Moreover, while providing women with evidence-based insights and educating women into the realities of breastfeeding are important strategies [13], we also found that encouraging women to have a ‘first feed’ could motivate women to sustain breastfeeding: the embodied experience could serve to dispel myths and reduce anxieties, leading to breastfeeding continuation.

### Clinical and practical implications

One of the key limitations of the peer support service concerned a lack of resources. A Cochrane review initially undertaken in 2012 [20] and updated in 2016 [21] identified that in order for additional breastfeeding support to be effective, it needs to be proactive, delivered face-to-face, and of sufficient intensity. These factors were echoed by our participants who highlighted the need for sufficient postnatal support that includes home visits (to build relationships and observe feeds) and to encourage access to wider provision. The lack of funds to provide ‘sufficient’ peer support also highlighted in other areas of peer support delivery [53, 54].

While the use of theory within peer support interventions has been lacking, there have been two recent UK based breastfeeding peer support interventions that have used the BCW to inform intervention design and delivery. The first is the Mam-Kind study [35, 55] with peers trained in using motivational interviewing to enhance women’s motivation to breastfeed. The intervention offered proactive (up to 2 weeks) and then reactive support (up to 6 weeks) delivered in three areas of deprivation with low breastfeeding rates. Whilst a feasibility study, rather than an experimental design was undertaken, the findings highlight that peer supporters and women found the support to be acceptable, and the intervention could be delivered as intended, however, practical and intrapersonal issues were highlighted [55]. The second is the Assets-based infant feeding help Before and After birth (ABA) intervention [56]. Assets-based approaches focus on the positive capabilities of individuals rather than their needs or deficits, and aims to enhance self-esteem, resilience and social networks. In this study existing peers from two different areas were trained to complete a genogram (a family/friends infant feeding tree) with women, and to provide women with an assets leaflet that detailed local and national resources and opportunities for breastfeeding support. Proactive support was initiated in the antenatal period, and up to 5 months postnatally [56]. The results of a randomised feasibility study to explore the acceptability and outcomes of the ABA intervention are forthcoming.

The Mam-Kind and ABA interventions offer a positive move in the use of and assessing the efficacy of different theoretical frameworks within breastfeeding peer support intervention designs. However, we consider that our findings provide useful contributions to the evidence base by offering more granular insights into how peer support can organically evolve. While we found small observed differences in breastfeeding rates following the introduction of the peer support service, similar to those reported by Scott et al. [57], the effects of individual components of the peer support service were not explicitly tested. Therefore, while the theoretically-informed insights into how peer support can influence women’s capabilities, motivation and opportunities appear to be important, we currently do not know what the ‘essential’ ingredients are. Further experimental research to rigorously test these positive delivery features and help determine their effectiveness on breastfeeding outcomes is needed.

### Strengths and limitations

The study’s strengths include different data collection methods (surveys, qualitative interviews) to gain an in-depth assessment of how the peer support service worked in practice. The longitudinal nature of the evaluation allowed us to assess how the service had adapted over time and to illuminate challenges that were less amenable to resolution. The lack of a theoretical framework such as the BCW to inform the design of the service is an obvious limitation; however, we believe the use of the BCW as an interpretive scaffold provides useful insights to inform the design of future peer support interventions. Other limitations are the relatively low participant numbers in some of the surveys, the lack of demographic data for some of the surveys (to reduce burden on participants and maximise responses) and the lack of younger women (aged under 30 years) among the survey respondents (where demographic data was collected). Unfortunately, while we were unable to collect denominators to provide antenatal, hospital and breastfeeding group survey completion rates, the number of women who received a home visit/completed a community survey was rather low. Further methods to increase completion, such as a thank-you voucher, or reminders should be included in similar studies.

## Conclusions

We used the three layers of the Behaviour Change Wheel as an analytical lens to interpret existing evaluation data to understand how an infant feeding peer support service had been operationalised, and how different types of peer support influenced women’s breastfeeding experiences and behaviours. The policies (outer layer) wheel of the Behaviour Change Wheel helped identify how the service had evolved in a context of good interdisciplinary relationships and partnership practices, in an area that had full BFI accreditation, used wide promotional methods and employed action planning cycles to adapt and enhance the support to meet service user needs. The second and inner layers of the Behaviour Change Wheel helped to identify how women’s physical and psychological capabilities were developed through information, practical support and demonstrations. Women’s motivation was enhanced through positive women-peer relationships, education on the health benefits of breastfeeding, and ongoing feedback. Opportunities related to a framework of flexible and proactive support provided within clinical and community venues. While some gaps and areas for development were highlighted, these theoretically informed insights into an organic and responsive peer support service help build the evidence base for breastfeeding peer support, and to identify positive delivery features for future testing.

## Supplementary information

**Additional file 1:** Interview topic guides. (DOCX 16 kb)

## Data Availability

Full/detailed anonymised survey responses, and additional anonymised quotes are available from the study authors.

## Abbreviations

A: Antenatal clinics
BCW: Behaviour change wheel
BFI: Baby Friendly Initiative
C: Community support
COM-B: Capabilities, opportunities, motivation – behaviour
G: Breastfeeding groups
H: Intrapartum support
UNICEF: United Nations Children’s Fund
