# Supplementary material for: Using the behaviour change wheel to explore infant feeding peer support provision; insights from a North West UK evaluation
Source: Int Breastfeed J. 2019 Sep 18;14:41. doi: 10.1186/s13006-019-0236-7 (PMC6749647; doi:10.1186/s13006-019-0236-7)
Supplement: Supplementary file 1 — Additional file 1: Interview topic guides. (DOCX 16 kb) [file 13006_2019_236_MOESM1_ESM.docx]

**Interview Guides**

***Health/Community Professionals***

- Knowledge of, experience and attitudes towards IFSS:
  - what do you know about the service (how information received, etc)?
  - how have you been involved with the service?
  - what has been your experience of the service (what is working well, not working well)?
  - how are peer supporters engaging with women (any issues of non engaging with certain women)?
- Impact of service on women, families and professionals (emotionally and socially)
  - what are your perceptions of the support being provided to women?
  - what if any, do you think are the benefits of this scheme (for women and families?)
  - what impact do you think the service has had on infant feeding, maternal wellbeing, parenting experiences?
  - what if any are the benefits of this service for professionals?
  - have there been any wider benefits of the service (i.e. in terms of how services are provided, training opportunities for staff, women forming networks, changing cultures around breastfeeding)?
- Recommendations/changes to practice
  - do there need to be any changes developments to how the service is offered/provided? (explore answer in terms of training, access, methods of engaging with women, communication, provision of services)

***Women***

To record women’s age, baby’s age, marital status, current as well as previous infant feeding method.

- Perceptions, attitudes and experiences of the peer support service:
  - how did you hear about the service?
  - what has been your involvement with the service (when was first contact made and how, frequency of contact, where contacts took place, etc)
  - what other advice/support around infant feeding have you received, i.e from professionals, family members?
  - what types of support did you receive (i.e. infant feeding, emotional support, help with ‘other’ issues)
  - what has been your experience of receiving support (what worked well, what didn’t work well, etc)
- Benefits of engagement
  - what have been the benefits (if any) of receiving support? (explore in relation to infant feeding, maternal wellbeing, parenting experiences, meeting new people/social opportunities, etc)?
  - what has been the impact of this support on your infant feeding experiences?
- Recommendations/changes to practice
  - do there need to be any changes developments to how the support is offered/provided? (explore answer in terms of access, methods of engaging with women, communication, provision of services, etc)

***Infant Feeding Leads/Commissioners/Peer Supporters (questions to focus around:***

Involvement in the IFSS?

How the service is working within practice?

Facilitators experienced – what is working well?

Barriers experienced – what is not working well?

Have there been any developments/changes to how the service is provided?

What else needs to happen to embed the service?
